# Multimodal deep learning models for early detection of Alzheimer’s disease stage

**DOI:** 10.1038/s41598-020-74399-w

**Published:** 2021-02-05

**Authors:** Janani Venugopalan, Li Tong, Hamid Reza Hassanzadeh, May D. Wang

**Affiliations:** 1 Department of Biomedical Engineering, Georgia Institute of Technology and Emory University, Atlanta, GA USA; 2 School of Computational Science and Engineering, Georgia Institute of Technology, Atlanta, GA USA; 3 School of Electrical and Computer Engineering, Georgia Institute of Technology, Atlanta, GA USA; 4 Winship Cancer Institute, Parker H. Petit Institute for Bioengineering and Biosciences, Institute of People and Technology, Georgia Institute of Technology and Emory University, Atlanta, GA USA

**Keywords:** Data integration, Data mining

## Abstract

Most current Alzheimer’s disease (AD) and mild cognitive disorders (MCI) studies use single data modality to make predictions such as AD stages. The fusion of multiple data modalities can provide a holistic view of AD staging analysis. Thus, we use deep learning (DL) to integrally analyze imaging (magnetic resonance imaging (MRI)), genetic (single nucleotide polymorphisms (SNPs)), and clinical test data to classify patients into AD, MCI, and controls (CN). We use stacked denoising auto-encoders to extract features from clinical and genetic data, and use 3D-convolutional neural networks (CNNs) for imaging data. We also develop a novel data interpretation method to identify top-performing features learned by the deep-models with clustering and perturbation analysis. Using Alzheimer’s disease neuroimaging initiative (ADNI) dataset, we demonstrate that deep models outperform shallow models, including support vector machines, decision trees, random forests, and k-nearest neighbors. In addition, we demonstrate that integrating multi-modality data outperforms single modality models in terms of accuracy, precision, recall, and meanF1 scores. Our models have identified hippocampus, amygdala brain areas, and the Rey Auditory Verbal Learning Test (RAVLT) as top distinguished features, which are consistent with the known AD literature.

Deep-learning (DL) has shown tremendous potential for clinical decision support for a variety of diseases, including diabetic retinopathy^1,2^, cancers^3,4,^ and Alzheimer’s disease (for imaging analysis)^5–7^. The major strength of DL over other shallow learning models is their ability to learn the most predictive features directly from the raw data given a dataset of labeled examples. DL has shown improvement over shallow learning for single data modality such as images^8,9^, electronic health records (EHRs)^10,^ and SNPs^11^. DL techniques also facilitate the training and prediction in the presence of partial data^12^. In this study, we develop a novel DL architecture for clinical decision support that predicts the Alzheimer’s disease (AD) stage using multi-modality data (images, clinical data, and genetic information).

AD is the most common neurodegenerative disorder and the 6th leading cause of death in the United States^13,14^. The world-wide disease burden of AD is projected to reach $2 trillion by 2030^15^, which necessitates early detection. Despite extensive research and advances in clinical practice, less than 50% of the AD patients are diagnosed accurately for their pathology and disease progression based on their clinical symptoms^13^. The most conclusive evidences for AD are the presence of amyloid plaques and neurofibrillary tangles in histopathology. However, the early onset of AD is not correlated with the presence of plaque but with synaptic and neuronal loss^16^.

Research on data and data mining strategies from AD initiative^17–19^ are ongoing to improve our understanding of the underlying disease processes. AD biomarkers including clinical symptoms^20^ (such as dementia, memory loss), neurological tests and scores such as MMSE scores are augmented with imaging, genetic, and protein biomarkers^21–26^. Most of these studies identify biomarkers using a single-modality data, which restricts a holistic assessment of AD disease progression. There have been AD multi-modal analyses that combine various imaging modalities^27–32^ such as structural MRI (T1 weighted, T2 weighted), fMRI, positron emission tomography (PET)^33,34^, and imaging genetics^35^. In addition, genetics have been used with clinical data to augment data labels and phenotypes. Besides shallow learners, DL models such as auto-encoders^8^ and deep-belief networks^36^ (Supplementary Table A1) have been used for PET and MRI image data fusion with improved prediction.

In this study, we further the multi-modal AD data fusion to advance AD stage prediction by using DL to combine imaging, EHR, and genomic SNP data for the classification of patients into control (CN), MCI, and AD groups. We use stacked de-noising auto-encoders for EHR and SNP data respectively, and novel 3D convolutional neural networks (CNNs) to train MRI imaging data. After the networks are separately trained for each data modality, we combine them using different classification layers, including decision trees, random forests, support vectors machines (SVM), and k-nearest neighbors (kNN). We demonstrate the performance of our integration models using the ADNI^37^ dataset that contains SNP (808 patients), MRI imaging (503 patients), and clinical and neurological test data (2004 patients).

Despite superior performance in clinical decision support using multiple data types, a major drawback for widespread adoption of DL models for clinical decision making is the lack of well-defined methods for interpreting the deep models. We address this challenge by developing novel perturbations and a clustering-based approach for finding the top features contributing to the decision.

In this article, we report the major contributions for the AD stage prediction as follows:

- Novel DL architectures outperform shallow learning models;
- Multi-modality data analysis with DL outperforms single-modality DL models; and
- Novel interpretable DL methods are capable of extracting top performing features.

## Data description

**Figure 1:**
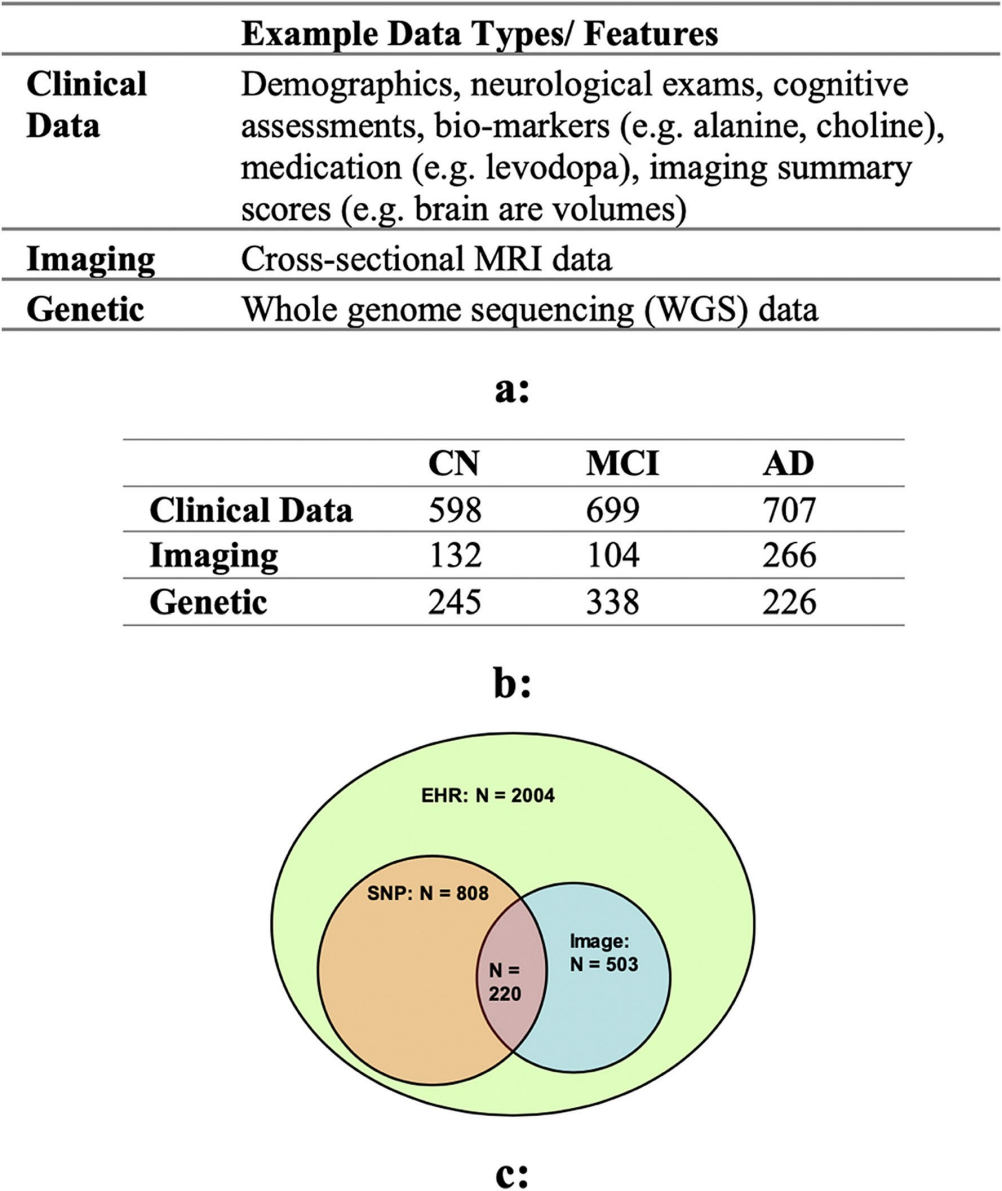
(**a**) Description of ADNI data. Clinical data consists of demographics, neurological exams and assessments, medications, imaging volumes, and biomarkers. (**b**) Number of patients by modality and disease stage. (*CN* controls, *MCI* mild cognitive disorder, and *AD* Alzheimer’s disease). 220 patients have all the three data modalities, 588 patients have SNP and EHR, 283 patients have imaging and EHR, the remaining patients have only EHR data.

This article uses Alzheimer’s Disease Neuroimaging Initiative* (ADNI) database (adni.loni.usc.edu)^37^ data for the analysis. ADNI aims to test whether serial MRI, PET, biological markers, and clinical and neuropsychological assessments can be combined to measure the progression of MCI and early AD. ADNI data repository contains imaging, clinical, and genetic data for over 2220 patients spanning over four studies (ADNI1, ADNI2, ADNI GO, and ADNI3). Our study focuses on ADNI1, 2 and GO because ADNI 3 is an ongoing study expected to end in 2022. The data is currently being released in phases with limited availability for unprocessed imaging data and no genetic data yet. The imaging data (ADNI1, 2 and GO) consists of MRI and PET images, of which we use cross-sectional MRI data corresponding to the baseline screenings from ADNI1 (503 patients). The data publisher has standardized the images to eliminate the non-linearities caused by the scanners from different vendors. In this study, we used the cross-sectional MRI data, consisting of 9108 voxels per patient distributed over 18 slices, with each slice having 22 × 23 voxels. For clinical or EHR data, we use 2004 patients (ADNI1, ADNI2, and ADNI GO) data from the clinical tests (e.g., memory tests, balance tests, and cognitive tests), medication data (e.g., usage of levodopa), imaging score summaries (e.g., levels of fluorodeoxyglucose (FDG) from PET, brain volumes from MRI), patient demographics (e.g., age and gender), and biochemical tests. The genetic data consists of the whole genome sequencing (WGS) data from 808 ADNI participants (at the time of sequencing, 128 with AD, 415 with MCI, and 267 controls) by Illumina’s non-Clinical Laboratory Improvement Amendments (non-CLIA) laboratory at roughly 30–40 × coverage in 2012 and 2013. The resulting variant call files (VCFs) have been generated by ADNI using Broad best practices (Burrows-Wheeler Aligner (BWA) and Genome Analysis Toolkit (GATK)-haplotype caller) in 2014. We use a total of 2004 patients in this study, with all 2004 patients have clinical data, 503 patients have imaging data, and 808 patients have genetic data (Fig. 1). For participants with multiple visits, we use the diagnosis from patient’s last visit. As shown in Fig. 1c, 220 patients have all three data modalities, 588 patients have SNP and EHR, 283 patients have imaging and EHR, the remaining patients have only EHR data.

## Study design for novel DL and multi-modality data analysis

As mentioned above, we use data from imaging (503 MRI images), SNP (808 patients) and the EHR (2004 patients) to predict AD stages. For each single data modality, we first demonstrate the superiority of deep models over shallow models such as kNN, one-vs-one coding SVM, random forests, and decision trees. The SNP and EHR features for shallow models and DL are the same. For imaging, when using DL, we apply multi-slice 3D voxels directly, while for shallow learners, we extract expert crafted features derived from the 3D voxels.

**Figure 2:**
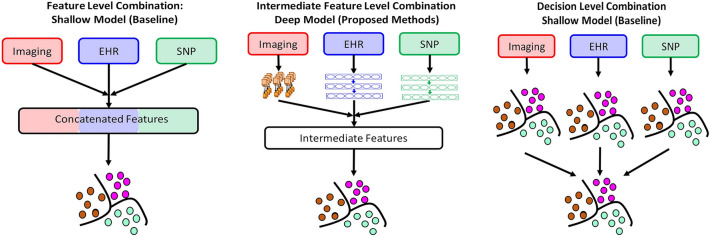
Deep model for data integration compared with shallow models of data integration. (**a**) Feature level integration on shallow models, where the features are concatenated before passing into shallow models. (**b**) Deep intermediate feature level integration where the original features are transformed separatelyusing deep models prior to integration and prediction. (**c**) Decision level integration where voting is performed using decisions of individual classifiers. In this study, we comparee the performance of deep intermediate level integration against shallow feature and decision levels integrations for the prediction of Alzheimer’s stages.

**Figure 3:**
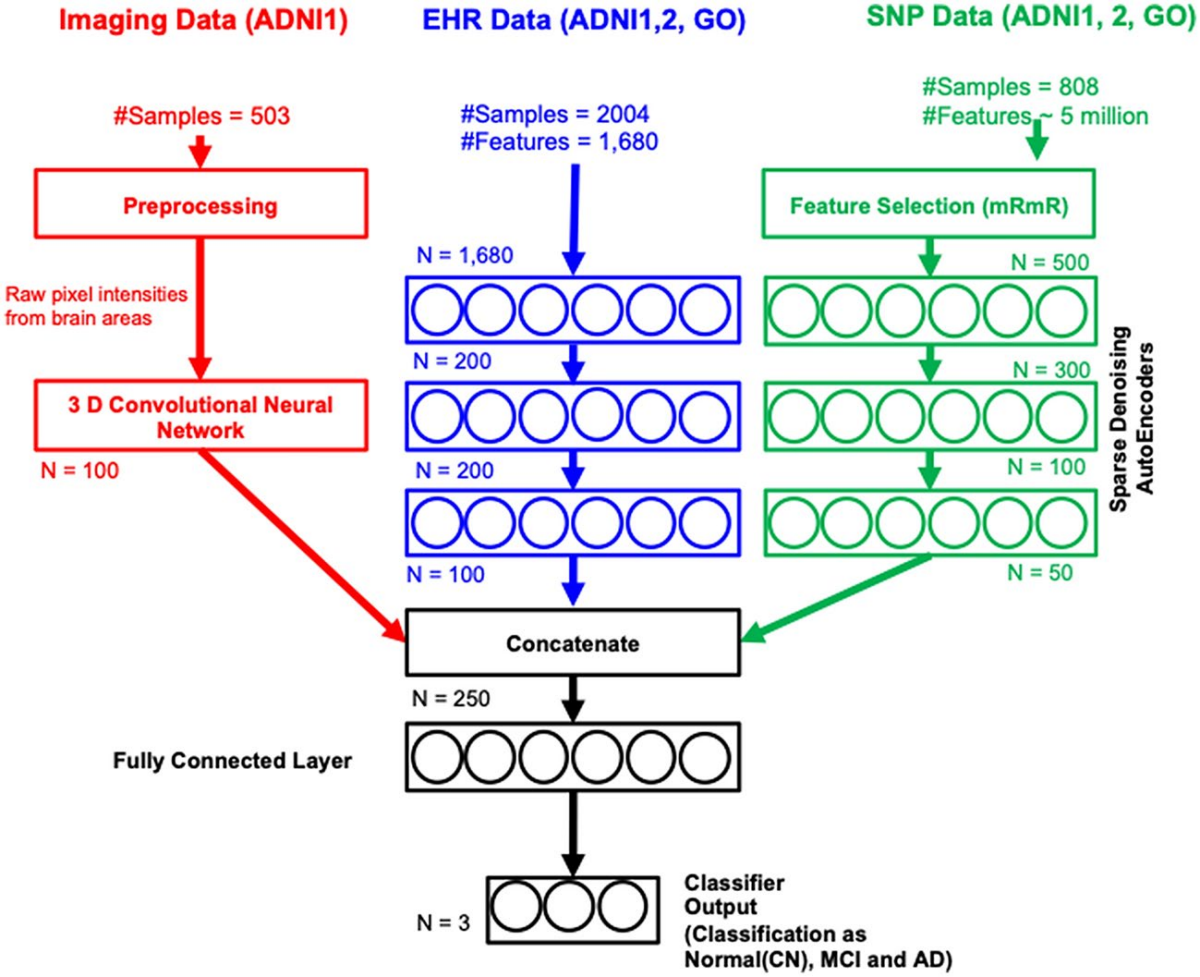
Intermediate-feature-level combination deep models for multimodality data integration for clinical decision support. Data from diverse sources, imaging, EHR and SNP are combined using novel deep architectures. 3D convolutional neural network architectures used on 3D MR image regions to obtain intermediate imaging features. Deep stacked denoising autoencoders are used to obtain intermediate EHR features. Deep stacked denoising autoencoders are used obtain intermediate SNP features. The 3 types of intermediate features are passed into a classification layer for classification into Alzheimer’s stages (CN, MCI and AD).

Regarding AD staging, only EHR has three-stage classes CN, MCI, and AD. SNP expression does not vary between MCI and AD^38^, and only has CN vs AD/MCI prediction. On images, patients with early MCI were structurally similar to CN, and those from patients with late MCI were structurally similar to AD. As such, for imaging, only CN and AD (as seen in Ref.^39^) are used for staging assessment. Thus, combining all three modalities can help us significantly improve AD staging prediction accuracy. As shown in Figs. 2 and 3. we have developed three data fusion strategies: (i) Feature-level combinations using shallow models, (ii) Intermediate-feature-level combinations using deep models, and (iii) Decision-level combinations using shallow models.

Feature-level combinations are performed through direct concatenation of the data modalities using shallow learners (Fig. 2). The intermediate-feature-level combinations are performed by extracting intermediate features using DL, followed by concatenating and passing through a classification layer (more details are provided in methods and supplement). Decision-level combinations are performed by voting on the single-modalities. We test shallow models such as kNN, one-vs-one coding SVM, random forests, and decision trees for decision-level combinations and present the best performing model. For the intermediate-feature-level models (Fig. 3), we evaluate four combinations, (i) EHR + imaging + SNP, (ii) EHR + imaging, (iii) EHR + SNP, and (iv) imaging + SNP. For all combinations except imaging + SNP, we perform three-stage classification (CN, AD, and MCI). For imaging + SNP we perform classification into AD vs CN.

All above-mentioned cases are evaluated using an internal cross-validation and an external test set. We first remove 10% of the data as an external test set. On the remaining 90%, we perform tenfold cross-validation, with 81% of the total data being used for training and 9% for internal cross-validation. The internal cross-validation data set is used to optimize the model.

## Results for novel DL and multi-modality data analysis

We report the ADNI results for both the internal cross-validation partition and the external test dataset. For each of the DL models, or the baseline shallow models, we use mean values of accuracy, precision, recall, and meanF1 scores as metrics to show the superiority of deep models for single-modalities and the improvements gained from data integration.

### 3D convolutional neural network (DL) is superior to shallow models on imaging MRI data

One patient’s imaging data consists of 9108 3D voxels of dimension 22 × 23 × 18, corresponding to each of the five selected brain areas.

**Figure 4:**
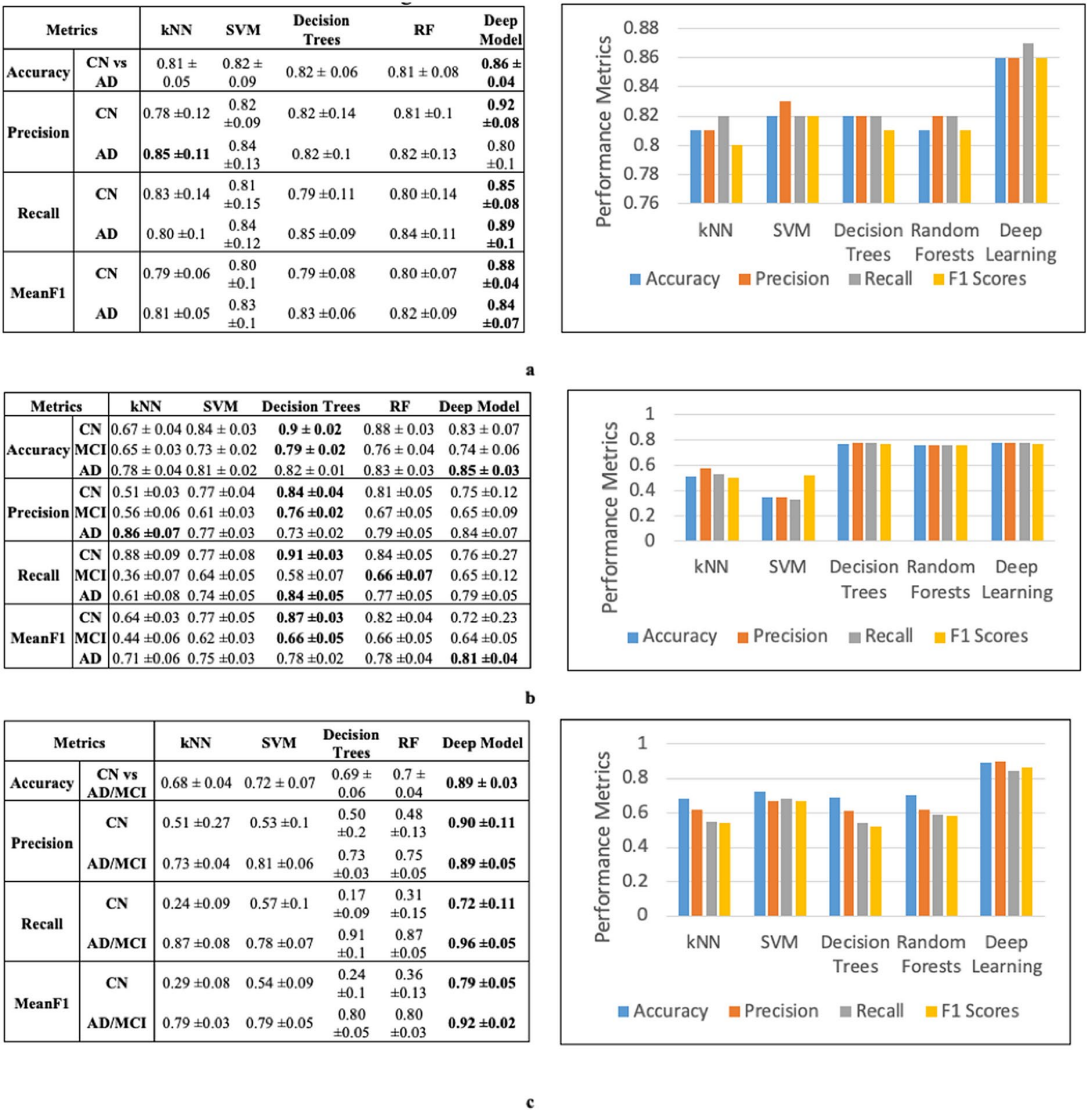
Internal cross validation results for individual data modality to predict Alzheimer’s stage (**a**) Imaging results: deep learning prediction performs better than shallow learning predictions (**b**) EHR results: deep learning outperforms shallow models kNN and SVM and is comparable to decision trees and random forests (**c**) SNP results: deep learning outperforms shallow models. The kNN, SVM, RF and decision trees are shallow models. (*kNN* k-Nearest Neighbors, *SVM* support vector machines, and *RF* random forests).

The number of nodes in DL models for the first-level fully connected layers = 5 × 20 = 100, and the number of nodes for the second level fully connected layer is 20. The results (Fig. 4a) indicate that the CNN based imaging models outperform shallow models and give the best precision and meanF1 scores.

### Deep autoencoder model is comparable to shallow models on EHR data

EHR data consists of 2004 patients with 1680 normalized features per patient, which we use to classify the patients into AD, MCI, and CN (three class). We use a three-layer auto-encoder with 200, 100 and 50 nodes each. The deep networks are trained using Adam with a max epoch count (repetition of DL network training on the entire dataset to allow adequate training) of 25. After hyperparameter optimization, the regularization coefficients for initial training is fixed at 0.03 and those for fine tuning at 0.03. The dropout probability is set to 0.6 for all the layers. The results (Fig. 4b) indicate that the autoencoders outperform shallow models such as kNN and SVM, and they are comparable to decision trees and random forests.

### Deep autoencoder model is superior to shallow models for SNP data

Processed SNP data consists of 808 patients with 500 features (each with levels 1, 2, 3), which we use to classify the patients into AD/MCI vs CN (two class). The auto-encoder network consists of three hidden layers with 200, 100 and 50 nodes each. Using Adam optimization and a max epoch count of 30, the best performing models have regularization coefficients for initial training as 0.03 and those for fine tuning at 0.06. The corruption (dropouts) is 0.6 for each layer. The results (Fig. 4c) indicate that the auto-encoder models outperform all the baselines models.

### Results for multi-modality classification

The intermediate features generated from the single-modality deep-models are concatenated and passed to an additional classification layer for integration.

#### Combination of all 3 modalities: (imaging + EHR + SNP): deep model outperforms shallow models

**Figure 5:**
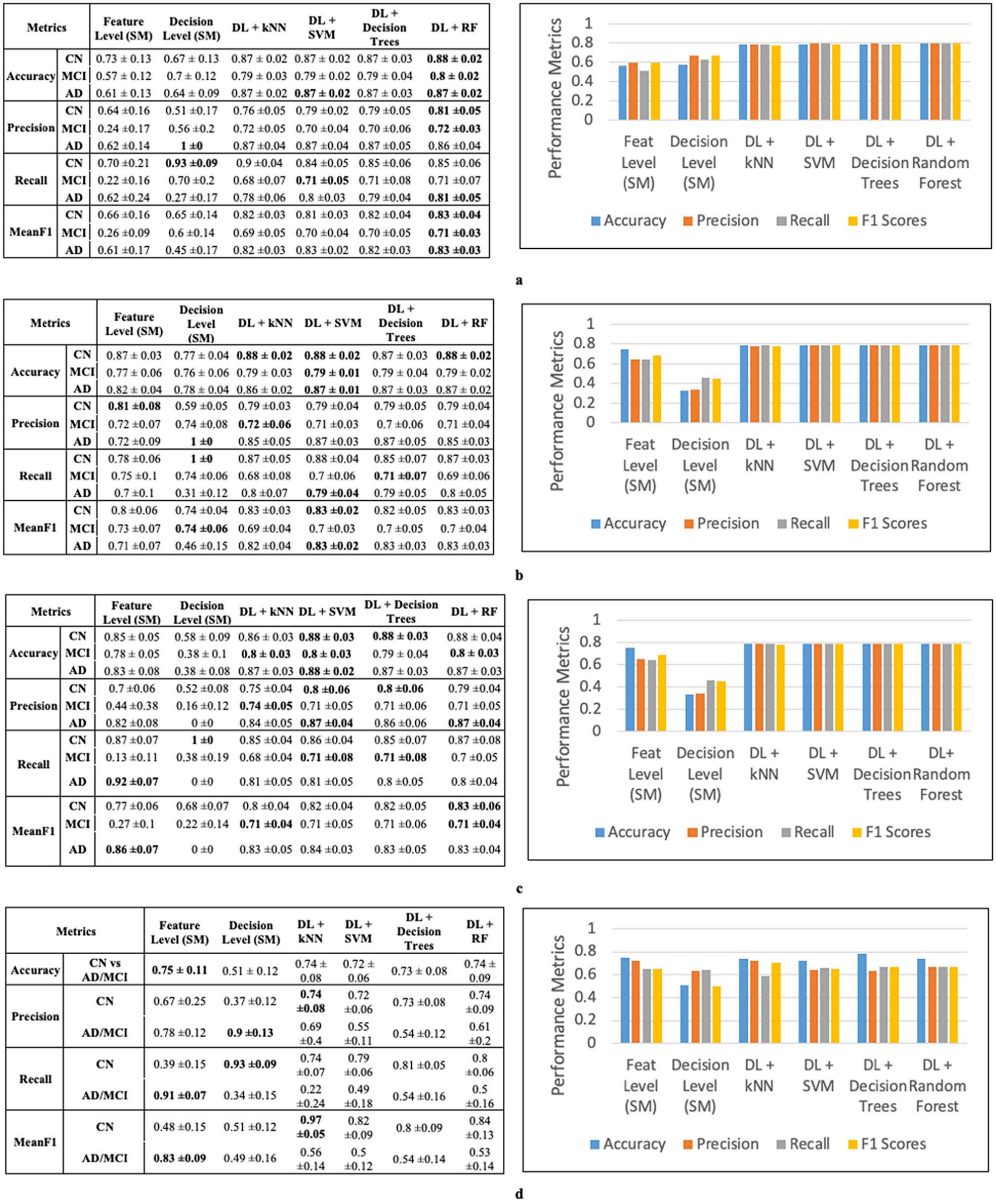
Internal cross validation results for integration of data modalities to predict Alzheimer’s stage (**a**) Imaging + EHR + SNP. Deep learning prediction performs better than shallow learning predictions (**b**) EHR + SNP Deep learning prediction performs better than shallow learning predictions (**c**) Imaging + EHR deep learning prediction performs better than shallow learning predictions (**d**) Imaging + SNP results. Shallow learning gave a better prediction than deep learning due to small sample sizes. (*kNN* k-Nearest Neighbors, *SVM* support vector machines, *RF* random forests, *SM* shallow models, and *DL* deep learning).

When a particular modality is not available, we mask it as zeros when using DL. The intermediate features from the three modalities are passed to the classification layer. We test kNN, decision trees, random forests, and support vectors machines as alternatives for the classification layer. Internal cross-validation (CV) accuracy (Fig. 5a) using deep models followed by random forests as the classification layer are the best. Deep models for the combination of the three modalities outperform single-modalities DL. In addition, during combination deep model outperforms shallow models such as feature-level and decision-level for both CV and external test sets (Table 1).

**Table 1:** Features extraction from deep models and comparison of internal validation results with external test result.

	Models	Biomarkers extracted	Internal cross validation performance	External test performance
EHR (deep models) (CN, MCI, AD)	Regularization coefficients (0.03, 0.03) Dropouts (0.6, 0.6, 0.6) Layer sizes (200, 100, 75)	Memory summary score RAVLT memory test (learning) RAVLT memory test (learning) baseline Neurophysiological battery (AVTOT 6 trials) Metabolomics marker (pe.P.16.0 22.6)	Accuracy: 0.78 ± 0.03 Precision: 0.78 ± 0.04 Recall: 0.78 ± 0.05 F1 Scores: 0.77 ± 0.04	Accuracy: 0.76 Precision: 0.76 Recall: 0.77 F1 Scores: 0.76
Imaging (deep models) Prediction (CN, AD)	Highest on validation (Dropout-0.5, Batch size 5 , Layer size(20), # areas = 5) Highest on external test (SVM kernel = linear)	Left hippocampus Right hippocampus Right superior temporal Right amygdala Left amygdala	Accuracy: 0.86 ± 0.04 Precision: 0.86 ± 0.04 Recall: 0.87 ± 0.04 F1 Scores: 0.86 ± 0.04	Accuracy: 0.84 Precision: 0.83 Recall: 0.83 F1 Scores: 0.83
SNP (deep models) Prediction (CN, MCI/AD)	Regularization coefficients (0.03, 0.03), Dropouts (0.6, 0.6, 0.6) Layer sizes (200, 100, 50)	Gene1 location 207782707 Gene1 location 55342929 Gene10 location 106979076 Gene10 location 50858045 Gene11 location 121493001	Accuracy: 0.89 ± 0.03 Precision: 0.9 ± 0.04 Recall: 0.84 ± 0.03 F1 Scores: 0.86 ± 0.04	Accuracy: 0.66 Precision: 0.66 Recall: 0.57 F1 Scores: 0.53
EHR + SNP + Imaging (deep models) Prediction (CN, MCI, AD)	Regularization coefficients (0.03, 0.03) Dropouts (0.6, 0.6, 0.6) Layer sizes (200, 100, 50) Random Forest Trees = 31	Voxel based morphometry Angular left Biomarker (PtdCho 16:0/18:1) MR volumes posterior limb of internal capsule including cerebral peduncle right Biomarker (PC ae C40:5) Biomarker (PC ae C42:4)	Accuracy: 0.79 ± 0 Precision: 0.79 ± 0.07 Recall: 0.79 ± 0.07 F1 Scores: 0.79 ± 0.07	Accuracy: 0.78 Precision: 0.77 Recall: 0.78 F1 Scores: 0.78
EHR + SNP (deep models) Prediction (CN, MCI, AD)	Regularization coefficients (0.03, 0.03) Dropouts (0.6, 0.6, 0.6) Layer sizes (200, 100, 50) Random Forest Trees = 31	Biomarker (Asymmetric dimethylarginine) Neuropsychological Battery (AVERR total intrusions) Neuropsychological Battery (Auditory Verbal Learning Test Trial1) Memory Score Voxel based morphometry Amygdala left	Accuracy: 0.78 ± 0 Precision: 0.79 ± 0.07 Recall: 0.79 ± 0.09 F1 Scores: 0.79 ± 0.07	Accuracy: 0.78 Precision: 0.78 Recall: 0.79 F1 Scores: 0.78
EHR + Imaging (deep models) Prediction (CN, MCI, AD)	Regularization coefficients (0.03, 0.03) Dropouts (0.6, 0.6, 0.6) Layer sizes (200, 100, 50) Random Forest Trees = 31;	Biomarker (Asymmetric dimethylarginine) Neuropsychological Battery (AVERR total intrusions) Cortical Thickness Average of Right Pericalcarine Memory Score Voxel based morphometry Amygdala left	Accuracy: 0.79 ± 0 Precision: 0.79 ± 0.08 Recall: 0.79 ± 0.08 F1 Scores: 0.79 ± 0.07	Accuracy: 0.77 Precision: 0.76 Recall: 0.77 F1 Scores: 0.77
SNP + Imaging (shallow models) Prediction (CN, MCI/AD)	Random Forest Trees = 20	Mean GLCM 3 right superior temporal Sum GLCM 5 left amygdala Median GLCM 2 right hippocampus Gene10 location 108777098 Entropy intensity left hippocampus	Accuracy: 0.75 ± 0.11 Precision: 0.72 ± 0.16 Recall: 0.65 ± 0.09 F1 Scores: 0.65 ± 0.12	Accuracy: 0.63 Precision: 0.62 Recall: 0.57 F1 Scores: 0.56

Autoencoder models are preferred for EHR and SNP data and CNN for imaging data. For multi-modality models, the three modality models and two modality models (EHR + SNP, EHR + imaging gave the best prediction performance). For the multi-modality models, 3 or 4 combinations deep models outperformed shallow models.

#### Combination of SNP and EHR modalities: deep model outperforms shallow models

Internal CV accuracy of 0.78 ± 0 using deep models followed by random forests as the classification layer (Fig. 5b.) are the best. The deep models for EHR + SNP combinations outperform single-modalities DL. During combination, deep model outperforms shallow models such as feature-level combination models for both CV and external test sets (Table 1).

#### Combination of imaging and EHR modalities: deep model outperforms shallow models

Internal CV accuracy of 0.79 ± 0 using deep models followed by random forests and SVM as the classification layers (Fig. 5c) are the best. The deep models for EHR+ imaging combinations outperform single-modalities DL. In addition, during combination, DL model outperforms shallow models such as feature decision-level combination models for both CV and external test sets (Table 1). Random forests as the classification layer give the best performance on the external set.

#### Combination of imaging and SNP modalities: shallow model outperforms deep models

We perform two-class classification using a combination of SNP and imaging intermediate features (CN vs. AD/MCI). Internal CV accuracy of 0.75 ± 0.11, using feature-level combination models (Fig. 5d) is the best. However, the results on the external data are poor. The poor external validation can be attributed to having only 220 patients with both modalities of data.

## Discussion for novel DL and multi-modality data analysis

Our results suggest that the deep models outperform traditional shallow models for single-modalities. The shallow models typically require handcrafted features by experts. On the contrary, deep models can find the optimal set of features during training. In addition, deep models such as auto-encoders and CNNs can be used to perform unsupervised feature generation, and then to combine with a more sophisticated decision layer. This architecture enables the modeling of complex decision boundaries for multiclass classification problems^40^. Due to this property, deep models are particularly effective for the identification of MCI, which has been a clinical challenge in Alzheimer’s research due to small differences between the three groups. Because shallow models (except random forests) do not tolerate noisy and missing data or missing modalities well, for noisy data, DL gives the best performance for single-modalities.

The integration of multiple modalities improves the prediction accuracy (three of four scenarios). The deep models for integration also show improved performance over traditional feature-level and decision-level integrations. The DL’s superior performance is due to its ability to extract relationships amongst features from different modalities. When the dataset is very small (e.g., the combination of imaging and SNP), deep models do not perform well. The degraded performance could be caused by the lack of training data for networks. Overall, our investigations show that:

- For single-modality data (clinical, and imaging), the performances of DL models are always better than those of shallow models; and
- When using DL models, predictions by multi-modality data is better than those by single-modality data. The three best fusion set ups are: EHR + SNP, EHR + Imaging + SNP, and EHR + Imaging.

One bottleneck for our proposed DL-based data integration model is the small sample size of the ADNI dataset. To mitigate the small sample size challenge, we can utilize strategies such as transfer learning and domain adaptation^41^. For each data modality, we can adopt neural networks pre-trained on other similar datasets (e.g., CNN-based MRI/CT brain imaging classification model trained for other conditions). By composing our model with these pre-trained networks and their parameters, we can perform domain adaptation or fine-tune the network parameters using our labeled ADNI data. On the other hand, we can also perform an unsupervised feature representation learning for each data modality using publicly available data (e.g., The Cancer Genome Atlas (TCGA) dataset for SNPs).Our feature extraction step is performed independently for each modality in the current DL model, which is not trained end-to-end with the integration and classification step. One future direction is to enable end-to-end training and combine auto-encoders with other integration strategies besides feature concatenation^42,43^.

## Study design of novel feature extraction to assist in DL model interpretation

**Figure 6:**
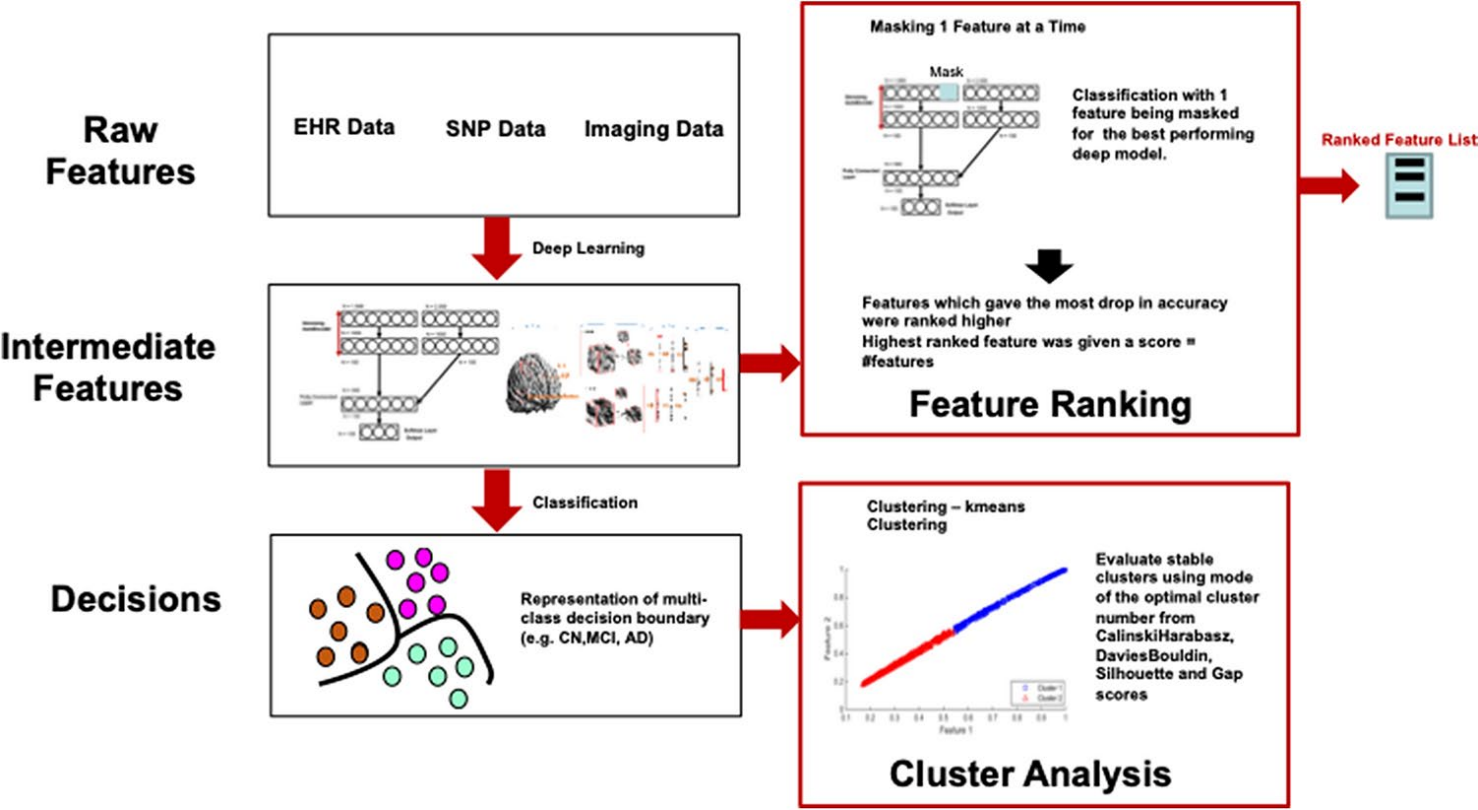
Feature extraction for deep model interpretation. Novel feature interpretation methodology where features are masked one at a time and the effect on the classification is observed. The feature which gives the highest drop in accuracy is ranked the highest. Once we ranked the features, we checked if the intermediate features picked associations different from raw data using cluster analysis. Deep models show associations which are different from shallow models, which accounts for superior performance.

Model interpretation is a major challenge for DL and is often considered as a barrier for real-world biomedical applications. Research has shown that the weights of deep models affect the results through several layers of combinations, hence do not yield clinically meaningful interpretation^44^. In this study, we develop a novel interpretation method where we mask one feature at a time and measure the drop-in accuracy (Fig. 6). The features that give the maximum drop in accuracy are ranked higher for feature extraction.

## Results and discussion of novel feature extraction to assist in DL model interpretation

The top EHR features (Table 1) include memory tests, imaging summary scores, and brain volumes. Changes to memory and brain volumes have been reported as AD biomarkers. Imaging markers such as involvement of limbic and cortical regions^45^, and changes in hippocampus volume and structure^46,47^ are known biomarkers in PET and MRI studies. SNP features picked chromosome 10, 4, 19, 1, and 5.

SNP + Imaging + EHR and SNP + EHR pick more EHR features (memory tests, metabolic markers and brain volume) which are known AD related features. EHR + Imaging pick EHR features including brain volumes, clinical dementia ratings, and metabolite markers. Imaging + SNP pick brain areas such as the hippocampus, and amygdala higher than SNP features.

In addition, we also cluster the intermediate features from EHR and SNP data using kmeans (Supplementary Information) to show associations in intermediate features. On plotting the clusters for intermediate and raw features, we find that the intermediate features generate better separation as compared to the original features. This indicates subtle relationships in intermediate features, which are picked by deep models (Supplementary Figs. A5, A6).

## Conclusions

Diagnosing patients with AD is challenging, and the prediction accuracy remains low for staging assessment. In this study, we report the potential of DL for multi-modal data fusion, including:

- Deep-models outperform shallow models for single-modality Alzheimer’s stage prediction.
- Novel DL framework for multi-modality data fusion outperforms single-modality DL.
- Novel perturbation and clustering-based feature extraction assisting DL model interpretations are capable of AD stage prediction.
- Application of 3D convolutional neural network architecture for MRI image data benefits the AD analysis.

Despite the improved performance, our study suffers from short-comings such as limited dataset sizes. In the future, we will test our models on a larger and richer dataset.

## Methods

In this study, we use DL models to perform multimodal data fusion (Fig. 3) (i.e. imaging, EHR and genomic SNP data) for classifying patients into CN, MCI, and AD groups. We use stacked de-noising auto-encoders for EHR and SNP, and 3D convolutional neural networks (CNNs) for MRI imaging data. After the networks are separately trained for each data modality, we apply decision trees, random forests, support vectors machines, and k-nearest neighbors to conduct integrated classification on AD staging.

### Data pre-processing

As mentioned above, ADNI dataset consists of clinical data, SNP data, and imaging data.

#### MRI imaging data

**Figure 7:**
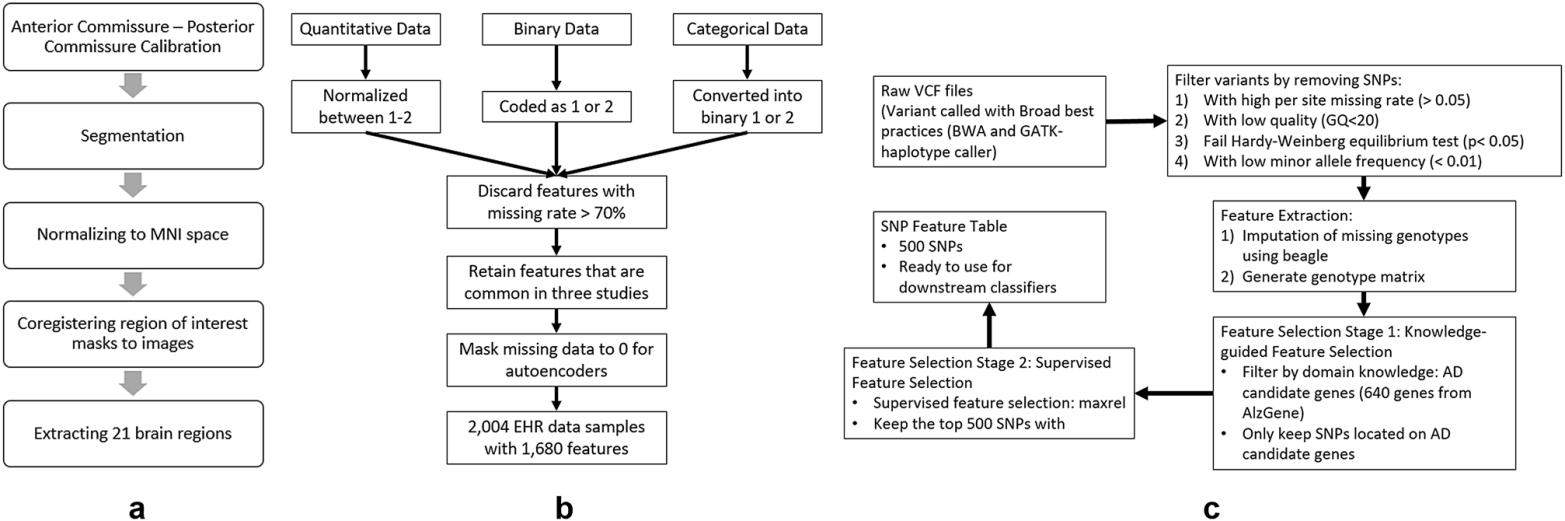
Data pre-processing pipeline for three data modalities: (**a**) Imaging data is first skull stripped, segmented into white matter, grey matter, and cerebrospinal fluid. Then the images are registered to a standard space, prior to extracting 21 brain regions using anatomical automatic labeling atlases. (**b**) Clinical data is normalized between 1–2 or encoded as 1–2. Then we discard features with values missing values > 70% to obtain 1680 features for 204 patients. (**c**) SNP data is first filtered, error corrected, feature selection using known genes and then followed by maximum relevance (maxrel) based methods, to obtain 500 SNPS for 808 patients.

We first preprocess the 3D images to filter noise, perform skull stripping, segment different types of brain tissue, normalize and co-register the images to MNI space (Fig. 7a)^48^. Following that, we extract 3D areas of 21 brain regions (associated with Alzheimer’s disease) including the right amygdala, left and right angular, left and right cerebellum, left and right Hippocampus, left and right occipital regions, and left and right superior temporal regions (Supplementary Information).

#### Clinical features

We extract 1680 common clinical features (quantitative real numbers, binary and categorical) from ADNI1, ADNI2, and ADNI GO. We normalize the quantitative data to the range 1–2, convert the categorical data into binary using one hot encoding., and finally, convert the binary data into values 1 or 2 (Fig. 7b).

#### Genetic data

Each subject has about ~ 3 million SNPs in the raw VCF file. We apply multiple filtering and feature selection steps (Fig. 7c) to eliminate SNPs with (i) low genotype quality, (ii) low minor allele frequency, (iii) high per-site missing rate and (iv) significant Hardy–Weinberg equilibrium p-value. After filtering, we apply a two-stage feature selection: (i) we retain SNPs that located on known AD-associated genes, (ii) we select 500 SNP features using minimum redundancy maximum relevance (mRMR)^49^ We chose mRMR as a feature selection method because it works well with categorical data (such the SNP data) and has been previously reported with genetic data^50^. mRMR was chosen over other wrapper-based techniques such as sequential feature selection due to computational costs. In the future we will investigate other filter-based feature selection methods such as correlation techniques, ANOVA, and relieFF in the future (Supplementary Information).

### Intermediate feature generation using single-modalities

We first perform feature extraction for each modality separately (Fig. 7), then we use DL for the generation of intermediate features. The intermediate features from EHR and SNP data are generated using auto-encoders and those of images are generated using 3D-convolutional neural networks. The intermediate features generated from each single-modality are subsequently used for multi-modal analysis. As a data-driven approach, DL’s performance heavily relies on a large amount of well-annotated training data. However, the ADNI dataset contains only a few thousand samples in total and even fewer samples with all three modalities. Thus, we use DL only for feature representation learning instead of end-to-end training.

#### Intermediate features for imaging data

First, we select the regions of interest and put them into a separate 3-dimensional convolutional neural network (Supplementary Fig. A2 in the supplementary material) with their weights shared across the CNN modules. CNN modules can extract higher level features from the abstraction of images to form concepts, that often correlate better with the targets. Each 3D CNN in the architecture above comprises ten 3D-convolutional kernels of size $5 \times 5 \times 5$ followed by pooling layers with pooling kernels of size $3 \times 3 \times 3$. After the pooling layer, we feed the pooled 3D images into Rectified Linear Unit (ReLU) non-linearities to learn complex features from the input modalities. We use volumetric batch normalization^51^ that is an effective regularizer for convolutional neural networks. Next, the feature maps generated by each 3D CNN are flattened and fed into separate fully connected layers with ReLU activation functions, followed by drop-out regularizers. We integrate the features generated from each modality and feed them into the second level fully connected layer and the corresponding drop-out layer. Finally, we use a softmax layer with a negative-log-likelihood loss function to train the imaging network.

We use the combined features generated from the first level fully connected layers as the intermediate features that are fed into our multi-modality DL models.

#### Intermediate features for EHR and SNP data using auto-encoders

We represent each patient data (EHR and SNP inputs to the feature learning algorithm) as a vector of length $m$(where $m$ is the number of features. Then, we pass this data through a two-layer stacked denoising auto-encoder network^52^ (Supplementary Fig. A3 in supplementary material) to obtain a high level representation of the patient data. Each auto-encoder layer takes an input $x$ of dimension $n \times d$, where $n$ is the number of training samples and $d$ is input dimensionality ($d = m$ for first layer). The input for each layer is first passed through an encoder to convert the input into a higher order representation of the data (1).

1 $$y = f\left( {Wx + b} \right),$$

where $f$ is an activation function such as sigmoidal or tanh, $\left[ {W,b} \right]$ are parameters to be trained. We then pass the mapped values $\left( y \right)$ through a decoder to obtain a representation of the input $(x$) (2).

2 $$\hat{x} = f\left( {W^{T} y + b^{\prime}} \right),$$

where $b^{\prime}$ needed to be trained, and the weights $W^{T}$ are tied with the encoder weights. We construct the network by stacking the trained encoder layers and implement denoising using dropouts, where a portion of the input values are masked (set to zero) to allow better generalization of the models in the presence of small and noisy training data. We perform training through back propagation by minimizing the average cross-entropy between the input and the reconstructed input data (3).

3 $$\left[ {W,b,b^{\prime}} \right] = \begin{array}{*{20}c} {\arg min } \\ {\left[ {W, b, b^{\prime}} \right]} \\ \end{array} - \mathop \sum \limits_{k = 1}^{a} \left[ {x_{k} \log\hat{x}_{k} + \left( {1 - x_{k} } \right) \log\left( {1 - \hat{x}_{k} } \right)} \right] ,$$

where $a$ is number of dimensions. Optimization is carried out using Adam optimization^53^ with a batch size of 3.

After the training of auto-encoder layers, we perform the network fine-tuning for each by adding a softmax layer that predicts the final class. The intermediate features are the output of the fine-tuned network after removing the softmax layer. The hyper-parameters in the model, such as the layer sizes, dropout parameters, and regularization coefficients (to prevent overfitting), are optimized using tenfold cross-validation.

#### Multimodal data integration

We propose data integration across modalities as a method for bridging the gaps in our understanding of disease processes and improve clinical outcome predictions and model performance. The data integration from different modalities can be performed at multiple levels (raw feature-level, intermediate feature-level, and decision-level)^54^ (Fig. 1). In this study, we integrate the intermediate features generated in the previous step using a concatenation layer followed by a classification layer to predict the AD stage (Fig. 3). We try k-nearest neighbors (kNN), decision trees, random forests, and support vectors machines (SVM) as alternatives for the classification layer. In the event any modality is missing for a specific patient, we mask the modality with zeros. This procedure minimizes the effect of missing values from propagating down the layers and hence allows prediction with some missing data. We evaluate our models using feature-level combinations and decision-level combinations as the baseline models.

## Supplementary information

Supplementary Information.
