# Supplementary material for: Multimodal deep learning models for early detection of Alzheimer’s disease stage
Source: Sci Rep. 2021 Feb 5;11:3254. doi: 10.1038/s41598-020-74399-w (PMC7864942; doi:10.1038/s41598-020-74399-w)
Supplement: Supplementary file 1 — Supplementary Information. [file 41598_2020_74399_MOESM1_ESM.docx]

**Title: Multimodal Deep Learning Models for Early Detection of Alzheimer’s Disease Stage**

Janani Venugopalan^a^, Li Tong^a^, Hamid Reza Hassanzadeh^b^, May D Wang^c^, Ph. D.

1. Department of Biomedical Engineering, Georgia Institute of Technology, Emory University Atlanta, GA, USA
2. School of Computational Science and Engineering, Georgia Institute of Technology, Atlanta, GA, USA
3. Departments of Electrical and Computer Engineering, Computational Science and Engineering, Winship Cancer Institute, Parker H. Petit Institute for Bioengineering and Biosciences, Institute of People and Technology, Georgia Institute of Technology and Emory University, Atlanta, GA, USA

**Corresponding author**

**May D. Wang, Ph.D.**

Departments of Biomedical Engineering, Electrical and Computer Engineering, Computational Science and Engineering, Winship Cancer Institute, Parker H. Petit Institute for Bioengineering and Biosciences, Institute of People and Technology,

Georgia Institute of Technology

Emory University, Atlanta, GA, USA

email: [maywang@bme.gatech.edu](mailto:maywang@bme.gatech.edu)

*Data used in preparation of this article were obtained from the Alzheimer’s Disease Neuroimaging Initiative (ADNI) database ([adni.loni.usc.edu](http://adni.loni.usc.edu/)). As such, the investigators within the ADNI contributed to the design and implementation of ADNI and/or provided data but did not participate in analysis or writing of this report. A complete listing of ADNI investigators can be found at: <http://adni.loni.usc.edu/wp-content/uploads/how_to_apply/ADNI_Acknowledgement_List.pdf>

**Supplementary Material**

| **SN** | **Deep-Method** | **Method Summary** | **Studies** | **Comments** |
| --- | --- | --- | --- | --- |
| 1 | Autoencoders | Unsupervised training of individual deep layers prior to stacking | MRI Imaging^1^ , EHR clinical decision support^2^ | Mainly single-modality studies using EHR data |
| 2 | Convolutional Neural Networks | Set of convolutional filters followed by fully connected layers | MRI Imaging^3^, Diabetic Retinopathy^4, 5^, Cancer^7^ | Single-modality studies and combination of images |
| 3 | Deep Belief Networks | Multiple layers of latent variables with connections between the layers but not between units within each layer | Multimodal imaging studies using different imaging modalities^8, 9^ | Multi-modal imaging modalities |

**Table A1: Deep Learning Methods in Alzheimer’s Literature. Tables gives a brief descriptions of the deep-methods, and study design for current Alzheimer’s Studies**

## **Data Preprocessing**

As mentioned in the main paper, we use the data from the Alzheimer’s Disease Neuroimaging Initiative*. Data used in the preparation of this article is obtained from the Alzheimer’s Disease Neuroimaging Initiative (ADNI) database (adni.loni.usc.edu). The ADNI has been launched in 2003 as a public-private partnership, led by Principal Investigator Michael W. Weiner, MD. The primary goal of ADNI has been to test whether serial magnetic resonance imaging (MRI), positron emission tomography (PET), other biological markers, and clinical and neuropsychological assessment can be combined to measure the progression of mild cognitive impairment (MCI) and early Alzheimer’s disease (AD).

## **Imaging Data**

We use cross-sectional MRI images from ADNI1 since those images have been standardized by the publisher of the data to eliminate the non-linearities caused by the scanners from different vendors. This step is critical to eliminate bias from the data collection, which could otherwise impact the deep-learning algorithm and contaminate the results. This gave a total of 503 patients in ADNI 1 with imaging data, with multiple images per patient. However they were collected at different times and have multiple labels assigned to them at different times. In our study, we perform a multi-modal analysis where the data from EHR and SNP are combined with the imaging data. For the non-imaging we had only the initial and final assessments. In addition, for genetic SNP data, since the gene expressions do not change over time, a study with time varying labels is not feasible. As a result, this in study design we restricted ourselves with the images at the baseline for early prediction.


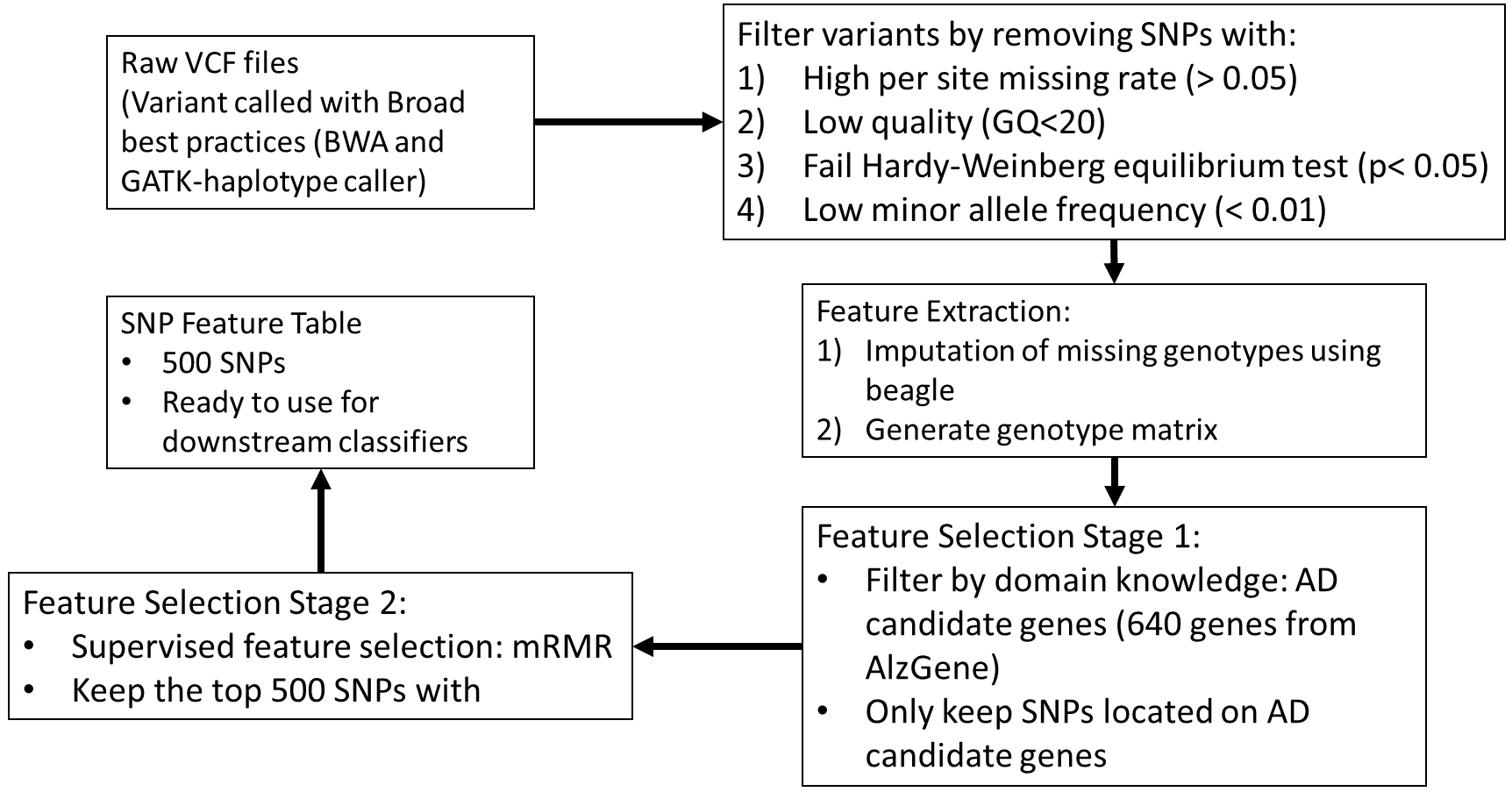


**Figure A1. Feature Extraction and Selection Pipeline for SNP Data. The SNP feature extraction pipeline starts from the raw VCF files downloaded from ADNI database. The raw VCF files are first filtered with multiple criteria to remove redundant and low-quality SNPs. We then impute the missing genotypes with the Beagle and generate a genotype matrix using VCFtools. The SNPs are further selected with 1) AD candidate genes and 2) mRMR supervised feature selection. We obtain a set of 500 SNPs after feature extraction and selection.**

As a first step, MR images are aligned according to the anterior commissure-posterior commissure, to correct for orientation errors, using the automatic registration toolbox (ART) (Klein 09). Next, we segment the images into the skull, cerebrospinal fluid (CSF), gray matter (GM) and white matter (WM) using the latest revision of the statistical parametric mapping toolbox (SPM12). The gray matter images are then normalized into the Montreal Neurological Institute (NMI) space. Then we select 21 regions of interests (ROI) masks from the AAL atlas that have been reported to be associated with the Alzheimer’s disease. In the next step, we register the GM images to each mask through affine transformation and subsequently, we crop a minimal bounding box that engulfs the corresponding masks from the registered images and thereby reducing the dimensions of the input images. Finally, we pad the selected sub-regions with zeros so that all the sub-regions across different masks have the same dimensions. Upon generation of 3D sub-regions from the normalized brains by co-registration of 21 masks whose association with Alzheimer has been proved in literature, we perform a recursive forward region selection strategy to choose the top regions that contribute the most to the prediction of the targets by training the overall model when the corresponding sub-models processing these regions are integrated into the model. The final included regions are the left and the right Hippocampus, the right superior temporal, and the right and the left Amygdala regions.

For comparing the performance of the CNN models, we extract baseline features including the brain volumes, voxel intensities, and texture based features. We extract features such as energy, entropy, and 13 Haralick texture features. Energy and entropy are calculated from multiwavelet transformation^6^. Haralick’s texture features are calculated using 64 gray-level co-occurrence matrix (GLCM) in 8 directions. Texture features calculated are the averaged texture features calculated across slices

**Figure A2. Genotype Matrix Generated using VCFtools. The four genotypes have been mapped to 0, 1, and 2 respectively.  The homozygous-dominant genotype (BB) is mapped to 0, the two heterozygous genotypes (Bb and bB) are mapped to 1, and the homozygous-recessive genotype (bb) is mapped to 2. During preprocessing the data are shifted between 1 to 4 and then normalized.**

## **EHR Data**

As mentioned in the main manuscript, the EHR data is quantitative, categorical or binary. The categorical data is converted to features using one-hot encoding, and quantitative features are normalized. This gives a total of 1,680 features.

**Figure A3: 3D Convolutional Neural Networks for 3D MRI Imaging Data (Deep Models). It is used to learn the filters that capture the interrelationship between brain regions of interest in all the three dimensions.**

## **SNP Data**

Each subject has about ~3 million SNPs in the raw VCF file generated by Broad best practice (bwa + ). However, not all of the SNPs detected are informative to the prediction of Alzheimer’s Disease. Some of the SNPs are called with a low confidence; some of these SNPs are rare SNPs that are only observed in a few subjects; some of the SNPs are correlated and we do not want to use all of them. So we need to apply quality control, domain knowledge and supervised feature selection to significantly reduce the number of features. The whole pipeline for feature extraction and selection is shown in Fig. A1.

For quality control, we first filter the SNPs with four criteria using vcftools ^10^:

- Hardy-Weinberg equilibrium (HWE) test for each site (p-values): remove SNPs with HWE $p<0.05$

| After mapping | CN  Label = 1 | | | MCI  Label = 2 | | | AD  Label = 3 | | |
| --- | --- | --- | --- | --- | --- | --- | --- | --- | --- |
| Original | Stable:  CN to CN  Label = 1 | Reversion:  MCI to CN  Label = 7 | Reversion:  AD to CN  Label = 9 | Stable:  MCI to MCI  Label = 2 | Conversion:  CN to MCI  Label = 4 | Reversion:  AD to MCI  Label = 8 | Stable:  AD to AD  Label = 3 | Conversion:  MCI to AD  Label = 5 | Conversion:  CN to AD  Label = 6 |

**Table A2: Rules for harmonizing the labels in ADNI dataset. Some of the data had labels indicating the disease progression as opposed to the actual labels. They were harmonized using directions from the ADNI website. The end state of the progression gave the final disease state of patient and was used in the study.**

- Genotype quality (GQ): remove SNPs with $GQ< 20$
- Minor allele frequency (MAF): remove sites with $MAF<0.01$
- Genotype value filtering: exclude sites on the basis of the proportion of missing data, remove sites with missing rate $> 0.05$

We have 7,610,182 SNPs after filtering with four criteria mentioned above. With the huge amount of SNPs (7,610,182 SNPs in total) after filtering in the previous feature extraction step, we use feature selection to reduce the number of SNPs for the perdition. We apply a two-stage feature selection. The first stage is an unsupervised feature selection utilizing domain knowledge. The second stage is a supervised feature selection using minimum Redundancy Maximum Relevance (mRMR) ^11^.

For the first stage, we utilize genes that are known to be related to Alzheimer’s Disease. In this step, we first download a list of all AD-related genes from AlzGene Database (<http://www.alzgene.org/>), which contains 680 genes in total. Then we search these genes in UCSC genome browser (<https://genome.ucsc.edu/>) and keep the 640 genes that matched NCBI RefSeq annotation. We extract these gene regions from RefSeq Annotation (gff file) in Bed format and use them to further filter the SNPs. We only retain the genes that are located in these regions. After feature selection using the domain knowledge, we have 143,504of SNPs.


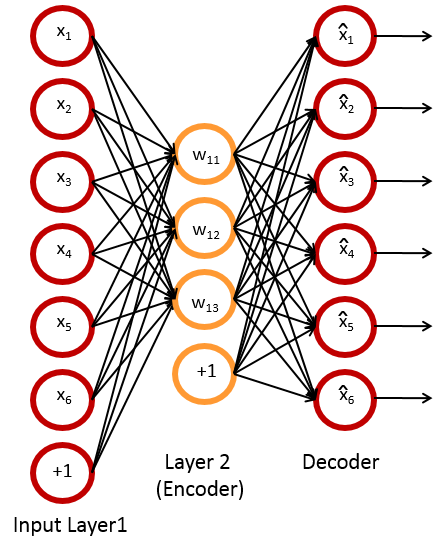


**Figure A4: Deep Learning Framework to Generate Intermediate Features using SNP and EHR Data. Deep autoencoder networks are used to generate intermediate features from preprocessed SNP and EHR data.**

After filtering with genes known to be related the AD, we use beagle ^12^ to impute the missing genotype. Then we use the vcftools to generate genotype matrix (Fig A2.) from the imputed vcf files. After filtering and imputation, we have a genotype matrix contains 0, 1, and 2 for all subjects, where the number represent that number of non-reference alleles. For the second stage, we apply the supervised feature selection method mRMR to further reduce the number of features. After mRMR feature selection, we have 500 SNP features retained.

## Matching of Labels

Most participants in ADNI1/GO/2 studies have multiple visits during the 48 month study period. The diagnosis of some of the patient's changes along time. However, in this study, we only focus on predicting the risk of developing AD instead of the progression of AD. To remove the influence of disease progression, we match the labels based on following rules:

- We only use the diagnosis of each patient’s last visit. This will give us the latest label of the patient.
- For labels representing the progression, we map them to following three corresponding labels: (1) control (CN), (2) mild cognitive impairment (MCI), and (3) Alzheimer’s disease (AD). The detailed label mapping rules are summarized in following Table A2.

## **Intermediate Feature Generation & Deep-Learning Models**

## **Intermediate Feature Generation Imaging Data**

Fig. A3. depicts the block diagram of the overall architecture of our imaging pipeline. Upon generation of 3D sub-regions from the normalized brains by co-registration of 21 masks whose association with Alzheimer has been proved in literature, we perform a recursive forward region selection strategy to choose the top regions that contribute the most to the prediction of the targets by training the overall model when the corresponding sub-models processing these regions are integrated into the model. The final regions include the left and the right Hippocampus, the right superior temporal, and the right and the left Amygdala regions.

## **Intermediate Feature Generation SNP and EHR Data**

As mentioned in the main manuscript, the EHR and SNP data is passed through a stacked autoencoder network. For each layer, the input is passed through a hidden layer (encoder) and the output is generated using a decoder (Fig. A4.). For stacked autoencoders, multiple encoders are stacked on top of each other. These are trained successively to produce its own input. The fine tuning of the entire network structure is performed using the labels in a supervised manner.

| Hyperparameters | Types | Values |
| --- | --- | --- |
| Regularization Coefficient | Initial Training | 0.03,0.01 |
|  | Fine Tuning | 0.003,0.001 |
| Dropout Value | For each Layer | 0, 0.2, 0.4, 0.6 |
| Hidden Layer Sizes | Layer 1 | 300, 200 |
|  | Layer 2 | 150, 100 |
|  | Layer 3 | 100,75, 50 |

**Table A3: Hyperparameters and Values of Hyper-parameters SNP and EHR Data. The validation data was used to fix the hyper0parameter value for deep autoencoders. The different values tried are given in table.**

## **Training & Hyper-Parameter Optimization for EHR and SNP Data**

As mentioned in the main manuscript, the data is divided into 10% for external testing, 90% for cross-validation (81% training, 9% validation). The hyperparameters to be optimized included coefficients of regularization, the dropouts, the sizes of each of the 3 hidden layers (total 8 hyperparameters). With 3 values (Table A3.) to search per coefficient, this gave a total of 6,541 combinations. Searching through all these combinations using grid search is prohibitive. In order to cut the search space, we use an alternating least square like approach to selecting the optimal hyperparameters. For each hyperparameter, we fix the values of the remaining hyperparameters and pick the value with the best accuracy on the validation dataset. We repeat this a few times until either the performance does not improve or the coefficients remain stable. For SNP data, we repeat the search 2 times and for EHR data we repeat the search 4 times to obtain the required hyperparameters.

| Data modality | Classification Task | Model | Hyper Parameters |
| --- | --- | --- | --- |
| EHR + SNP | Three-class | SVM | Kernel = ‘linear’ |
| EHR + Imaging | Three-class | Random forests | Trees = 20 |
| SNP + Imaging | Binary (AD+MCI vs. Control) | SVM | Kernel = ‘gaussian’ |
| EHR + SNP + Imaging | Three-class | Decision trees | Max splits (log 2), min leaf = 1 |

**Table A4: Parameters for Multi-Class Classifiers after Tuning (integration at feature level). Table gives the final hyper-parameter for the feature level combinations on the shallow classifiers. The shallow model hyper-parameters for the different comparisons were also tuned on the validation data.**

## **Baseline Classification Models- Shallow Models**

For multi-modality classification, we implement two integration strategies. One is integration at the feature-level, the other is the integration at the decision level.

## **Feature-level integration**

To realize feature-level integration, we first concatenate the feature vectors from each data modality for each sample and perform classification using decision trees. Then we use the concatenated features for classification. We tested kNN, decision trees, SVM, and random forests. The optimized parameters we use for the classifiers are listed in following Table A4.

## **Decision-level integration**

| Data modality | Classification Task | Model | Hyper parameters |
| --- | --- | --- | --- |
| EHR | Three-class | Decision trees | Max splits (log 2), min leaf = 7 |
| SNP | Binary (AD+MCI vs. Control) | SVM | Kernel = ‘linear’ |
| Imaging | Binary (AD vs. Control) | Decision trees | Max splits (sqrt), min leaf = 7 |

**Table A5: Parameters for Multi-Class Classifiers after Tuning (integration at decision level).. Tables gives the final hyper-parameter for the decision level combinations on the shallow classifiers. The shallow model hyper-parameters for the different comparisons were also tuned on the validation data**

For each data modality (SNP, EHR, and imaging respectively), we implement 10-fold cross-validation followed by a an external test set. First, we use the train and validation data to tune the following three parameters of the decision trees: the number of features to consider when looking for the best hyperparameters. To realize decision-level integration, we first calculate the class probabilities using single-modality classifiers built. Then we combine the decisions from all classifiers using simple majority voting.

## **Interpretation of Deep-Models**

As mentioned in the main paper, interpretability of deep-learning models is challenging. However, the Interpretability of deep-learning models constitutes a major challenge. In this work, we interpret the models by masking one feature at a time, the features that give the highest drop in accuracy are picked as the top features. We rank the feature with the highest drop as the top feature. For interpretation, we pick the model with the performance in each of the four integrations and for each single-modality model. For EHR autoencoder, the deep learning models pick memory tests. Integration models with all the modalities rank EHR features higher. The features for the combination models are different from those of the single-modalities. Models pick clinical dementia scores, metabolites, pre-existing conditions, and neurophysiological tests high.


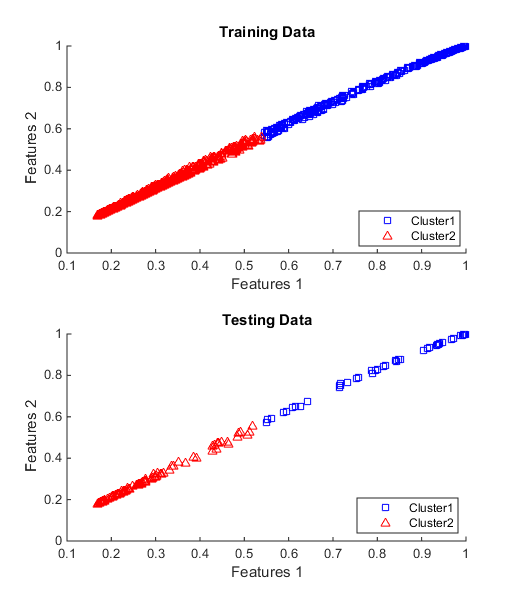

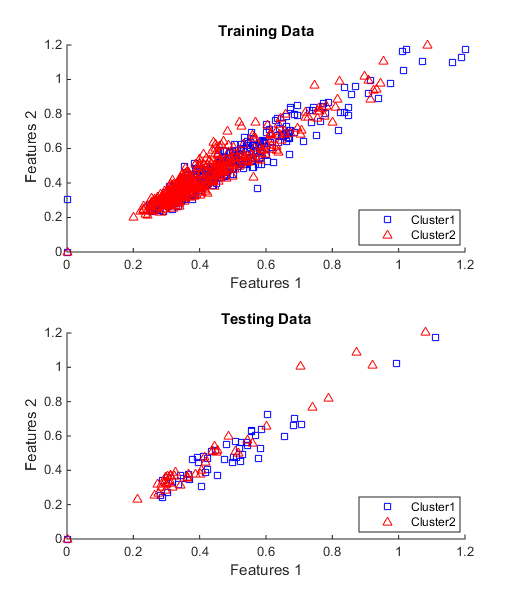


**A B**

**Figure A5: Novel Data Interpretation Results using Cluster Analysis to Demonstrate the Capability of Deep Models to Extract Data Relationships not Apparent in Raw EHR Data: A) intermediate features from EHR data readily separated into clusters. B) top ranked (using relieFF) EHR features did not separate into well-defined clusters.**

In order to test whether the intermediate features generated by the deep-models picked relationships in data, we use a clustering-based approach. We use kmeans to cluster the intermediate features from EHR and SNP. This was done to check if the intermediate features pick some subtle relationships not found in original data. The number of clusters and cluster centers are fixed using the training data, which we use to cluster the test data. The cluster number is evaluated using the mode of cluster number generated using Calinski Harabasz ^13^, Davies-Bouldin ^14^, silhouette ^15^ and gap scores ^16^. We use relieFF ^17^ feature selection to find the features that discriminated the clusters. The top features that consistently discriminate the clusters for both EHR and SNP data are reported. As mentioned in the main paper, on plotting the clusters for intermediate and raw features, we find that the intermediate features generate better separation as compared to the original features. This indicates subtle relationships in intermediate features, that are picked by deep-models (Fig A5., A6.).

**A B**

**Figure A6: Novel Data Interpretation Results using Cluster Analysis to Demonstrate the Capability of Deep Models to Extract Data Relationships not Apparent in Raw SNPData: A) intermediate features from SNP data readily separated into clusters. B) top ranked (using relieFF) SNP features did not separate into well defined clusters.**

## **Acknowledgments**

Data collection and sharing for this project was funded by the Alzheimer's Disease Neuroimaging Initiative (ADNI) (National Institutes of Health Grant U01 AG024904) and DOD ADNI (Department of Defense award number W81XWH-12-2-0012). ADNI is funded by the National Institute on Aging, the National Institute of Biomedical Imaging and Bioengineering, and through generous contributions from the following: AbbVie, Alzheimer’s Association; Alzheimer’s Drug Discovery Foundation; Araclon Biotech; BioClinica, Inc.; Biogen; Bristol-Myers Squibb Company; CereSpir, Inc.; Cogstate; Eisai Inc.; Elan Pharmaceuticals, Inc.; Eli Lilly and Company; EuroImmun; F. Hoffmann-La Roche Ltd and its affiliated company Genentech, Inc.; Fujirebio; GE Healthcare; IXICO Ltd.; Janssen Alzheimer Immunotherapy Research & Development, LLC.; Johnson & Johnson Pharmaceutical Research & Development LLC.; Lumosity; Lundbeck; Merck & Co., Inc.; Meso Scale Diagnostics, LLC.; NeuroRx Research; Neurotrack Technologies; Novartis Pharmaceuticals Corporation; Pfizer Inc.; Piramal Imaging; Servier; Takeda Pharmaceutical Company; and Transition Therapeutics. The Canadian Institutes of Health Research is providing funds to support ADNI clinical sites in Canada. Private sector contributions are facilitated by the Foundation for the National Institutes of Health (www.fnih.org). The grantee organization is the Northern California Institute for Research and Education, and the study is coordinated by the Alzheimer’s Therapeutic Research Institute at the University of Southern California. ADNI data are disseminated by the Laboratory for Neuro Imaging at the University of Southern California.

## **References**

1. Bhatkoti, P. & Paul, M. in Image and Vision Computing New Zealand (IVCNZ), 2016 International Conference on 1-5 (IEEE, 2016).

2. Miotto, R., Li, L., Kidd, B.A. & Dudley, J.T. Deep patient: an unsupervised representation to predict the future of patients from the electronic health records. *Scientific reports* **6**, 26094 (2016).

3. Payan, A. & Montana, G. Predicting Alzheimer's disease: a neuroimaging study with 3D convolutional neural networks. *arXiv preprint arXiv:1502.02506* (2015).

4. Ting, D.S.W. et al. Development and validation of a deep learning system for diabetic retinopathy and related eye diseases using retinal images from multiethnic populations with diabetes. *Jama* **318**, 2211-2223 (2017).

5. Gulshan, V. et al. Development and validation of a deep learning algorithm for detection of diabetic retinopathy in retinal fundus photographs. *Jama* **316**, 2402-2410 (2016).

6. Esteva, A. et al. Dermatologist-level classification of skin cancer with deep neural networks. *Nature* **542**, 115 (2017).

7. Suk, H.-I., Lee, S.-W. & Shen, D. Hierarchical feature representation and multimodal fusion with deep learning for AD/MCI diagnosis. *NeuroImage* **101**, 569-582 (2014).

8. Feng, X., Yang, J., Laine, A.F. & Angelini, E.D. in International Conference on Medical Image Computing and Computer-Assisted Intervention 568-576 (Springer, 2017).

9. Weng, S., Xu, X., Li, J. & Wong, S.T. Combining deep learning and coherent anti-Stokes Raman scattering imaging for automated differential diagnosis of lung cancer. *Journal of biomedical optics* **22**, 106017 (2017).

10. Suk, H.-I. & Shen, D. in Medical Image Computing and Computer-Assisted Intervention–MICCAI 2013 583-590 (Springer, 2013).

11. Liu, S. et al. Multimodal Neuroimaging Feature Learning for Multiclass Diagnosis of Alzheimer's Disease. *Biomedical Engineering, IEEE Transactions on* **62**, 1132-1140 (2015).

12. Suk, H.-I., Lee, S.-W., Shen, D. & Initiative, A.S.D.N. Deep sparse multi-task learning for feature selection in Alzheimer’s disease diagnosis. *Brain Structure and Function*, 1-19 (2015).

13. Schulam, P., Wigley, F. & Saria, S. in AAAI 2956-2964 (2015).

14. Suk, H.-I. & Shen, D. in International Conference on Medical Image Computing and Computer-Assisted Intervention 583-590 (Springer, 2013).

15. Choi, E., Bahadori, M.T. & Sun, J. Doctor ai: Predicting clinical events via recurrent neural networks. *arXiv preprint arXiv:1511.05942* (2015).

16. Zhou, J. & Troyanskaya, O.G. Predicting effects of noncoding variants with deep learning-based sequence model. *Nature methods* **12**, 931-934 (2015).

17. Ngiam, J. et al. in Proceedings of the 28th international conference on machine learning (ICML-11) 689-696 (2011).

18. , Vol. 2016 (Alzheimer's Disease Organization, 2016).

19. Alzheimer's, A. 2013 Alzheimer's disease facts and figures. *Alzheimer's & Dementia* **9**, 208-245 (2013).

20. Prince, M., Comas-Herrera, A., Knapp, M., Guerchet, M. & Karagiannidou, M. World Alzheimer report 2016: improving healthcare for people living with dementia: coverage, quality and costs now and in the future. (2016).

21. Perrin, R.J., Fagan, A.M. & Holtzman, D.M. Multimodal techniques for diagnosis and prognosis of Alzheimer's disease. *Nature* **461**, 916-922 (2009).

22. Grimmer, T. et al. Visual versus fully-automated analyses of FDG-and Amyloid-PET for prediction of dementia due to Alzheimer’s disease in mild cognitive impairment. *Journal of Nuclear Medicine*, jnumed. 115.163717 (2015).

23. Eskildsen, S.F. et al. Structural imaging biomarkers of Alzheimer's disease: predicting disease progression. *Neurobiology of aging* **36**, S23-S31 (2015).

24. Blennow, K. et al. Clinical utility of cerebrospinal fluid biomarkers in the diagnosis of early Alzheimer's disease. *Alzheimer's & Dementia* **11**, 58-69 (2015).

25. Chaves, R. et al. Efficient mining of association rules for the early diagnosis of Alzheimer's disease. *Physics in medicine and biology* **56**, 6047-6063 (2011).

26. de Haan, W. et al. Functional neural network analysis in frontotemporal dementia and Alzheimer's disease using EEG and graph theory. *BMC neuroscience* **10**, 101 (2009).

27. Hyman, B.T. et al. National Institute on Aging–Alzheimer's Association guidelines for the neuropathologic assessment of Alzheimer's disease. *Alzheimer's & Dementia* **8**, 1-13 (2012).

28. Mamikonyan, E. et al. Mild cognitive impairment is common in Parkinson's disease patients with normal Mini-Mental State Examination (MMSE) scores. *Parkinsonism & related disorders* **15**, 226-231 (2009).

29. Glodzik, L. et al. Alzheimer's disease markers, hypertension, and gray matter damage in normal elderly. *Neurobiology of Aging* **33**, 1215-1227 (2012).

30. Dubois, B. et al. Advancing research diagnostic criteria for Alzheimer's disease: the IWG-2 criteria. *The Lancet Neurology* **13**, 614-629 (2014).

31. Barnes, J. et al. Vascular and Alzheimer's disease markers independently predict brain atrophy rate in Alzheimer's Disease Neuroimaging Initiative controls. *Neurobiology of Aging* **34**, 1996-2002 (2013).

32. Doecke, J.D., Laws, S.M., Faux, N.G. & et al. BLood-based protein biomarkers for diagnosis of alzheimer disease. *Archives of Neurology* **69**, 1318-1325 (2012).

33. Dyrba, M., Grothe, M., Kirste, T. & Teipel, S.J. Multimodal analysis of functional and structural disconnection in Alzheimer's disease using multiple kernel SVM. *Human Brain Mapping* **36**, 2118-2131 (2015).

34. Shaffer, J.L. et al. Predicting Cognitive Decline in Subjects at Risk for Alzheimer Disease by Using Combined Cerebrospinal Fluid, MR Imaging, and PET Biomarkers. *Radiology* **266**, 583-591 (2013).

35. Dai, Z. et al. Discriminative analysis of early Alzheimer's disease using multi-modal imaging and multi-level characterization with multi-classifier (M3). *NeuroImage* **59**, 2187-2195 (2012).

36. Dyrba, M. et al. Predicting Prodromal Alzheimer's Disease in Subjects with Mild Cognitive Impairment Using Machine Learning Classification of Multimodal Multicenter Diffusion-Tensor and Magnetic Resonance Imaging Data. *Journal of Neuroimaging* **25**, 738-747 (2015).

37. Lorenzi, M. et al. Multimodal Image Analysis in Alzheimer’s Disease via Statistical Modelling of Non-local Intensity Correlations. *Scientific Reports* **6**, 22161 (2016).

38. Vogel, J.W. et al. Brain properties predict proximity to symptom onset in sporadic Alzheimer’s disease. *Brain* (2018).

39. Gray, K.R., Aljabar, P., Heckemann, R.A., Hammers, A. & Rueckert, D. Random forest-based similarity measures for multi-modal classification of Alzheimer's disease. *NeuroImage* **65**, 167-175 (2013).

40. Zhang, D., Wang, Y., Zhou, L., Yuan, H. & Shen, D. Multimodal classification of Alzheimer's disease and mild cognitive impairment. *NeuroImage* **55**, 856-867 (2011).

41. Wang, H. et al. Identifying disease sensitive and quantitative trait-relevant biomarkers from multidimensional heterogeneous imaging genetics data via sparse multimodal multitask learning. *Bioinformatics* **28**, i127-i136 (2012).

42. Mueller, S.G. et al. Ways toward an early diagnosis in Alzheimer’s disease: the Alzheimer’s Disease Neuroimaging Initiative (ADNI). *Alzheimer's & Dementia* **1**, 55-66 (2005).

43. Shen, L. et al. Genetic analysis of quantitative phenotypes in AD and MCI: imaging, cognition and biomarkers. *Brain imaging and behavior* **8**, 183-207 (2014).

44. Leandrou, S., Petroudi, S., Reyes-Aldasoro, C.C., Kyriacou, P.A. & Pattichis, C.S. Quantitative MRI Brain Studies in Mild Cognitive Impairment and Alzheimer's disease: A Methodological Review. *IEEE Reviews in Biomedical Engineering* (2018).

45. Mhaskar, H., Liao, Q. & Poggio, T. Learning functions: when is deep better than shallow. *arXiv preprint arXiv:1603.00988* (2016).

46. Pasupa, K. & Sunhem, W. in Information Technology and Electrical Engineering (ICITEE), 2016 8th International Conference on 1-6 (IEEE, 2016).

47. Che, Z., Purushotham, S., Khemani, R. & Liu, Y. Distilling knowledge from deep networks with applications to healthcare domain. *arXiv preprint arXiv:1512.03542* (2015).

48. Hampson, R.E. et al. Facilitation of memory encoding in primate hippocampus by a neuroprosthesis that promotes task-specific neural firing. *Journal of neural engineering* **10**, 066013 (2013).

49. Eickhoff, S.B. et al. A new SPM toolbox for combining probabilistic cytoarchitectonic maps and functional imaging data. *NeuroImage* **25**, 1325-1335 (2005).

50. Peng, H., Long, F. & Ding, C. Feature selection based on mutual information criteria of max-dependency, max-relevance, and min-redundancy. *IEEE Transactions on pattern analysis and machine intelligence* **27**, 1226-1238 (2005).

51. Ioffe, S. & Szegedy, C. Batch normalization: Accelerating deep network training by reducing internal covariate shift. *arXiv preprint arXiv:1502.03167* (2015).

52. Kingma, D. & Ba, J. Adam: A method for stochastic optimization. *arXiv preprint arXiv:1412.6980* (2014).

53. Ritchie, M.D., Holzinger, E.R., Li, R., Pendergrass, S.A. & Kim, D. Methods of integrating data to uncover genotype-phenotype interactions. *Nature Reviews Genetics* **16**, 85-97 (2015).

54. Haralick, R.M. Statistical and structural approaches to texture. *Proceedings of the IEEE* **67**, 786-804 (1979).

55. Danecek, P. et al. The variant call format and VCFtools. *Bioinformatics* **27**, 2156-2158 (2011).

56. Ding, C. & Peng, H. Minimum redundancy feature selection from microarray gene expression data. *J Bioinform Comput Biol* **3**, 185-205 (2005).

57. Howie, B.N., Donnelly, P. & Marchini, J. A flexible and accurate genotype imputation method for the next generation of genome-wide association studies. *PLoS Genet* **5**, e1000529 (2009).

58. Kryszczuk, K. & Hurley, P. in International Workshop on Multiple Classifier Systems 114-123 (Springer, 2010).

59. Davies, D.L. & Bouldin, D.W. A cluster separation measure. *IEEE transactions on pattern analysis and machine intelligence*, 224-227 (1979).

60. Rousseeuw, P.J. Silhouettes: a graphical aid to the interpretation and validation of cluster analysis. *Journal of computational and applied mathematics* **20**, 53-65 (1987).

61. Tibshirani, R., Walther, G. & Hastie, T. Estimating the number of clusters in a data set via the gap statistic. *Journal of the Royal Statistical Society: Series B (Statistical Methodology)* **63**, 411-423 (2001).

62. Robnik-Šikonja, M. & Kononenko, I. Theoretical and empirical analysis of ReliefF and RReliefF. *Machine learning* **53**, 23-69 (2003).
